# Electrophysiology and fluorescence to investigate cation channels and transporters in isolated plant vacuoles

**DOI:** 10.1007/s44154-022-00064-z

**Published:** 2022-10-01

**Authors:** Antonella Gradogna, Armando Carpaneto

**Affiliations:** 1 Institute of Biophysics, National Research Council, Via De Marini 6, 16149 Genoa, Italy; 2 Department of Earth, Environment and Life Sciences (DISTAV), University of Genoa, Viale Benedetto XV 5, 16132 Genoa, Italy

**Keywords:** Patch-clamp, Fura-2, BCECF, TPC channels, Potassium, Calcium, NHX antiporters, Proton

## Abstract

The plant vacuole plays a fundamental role in cell homeostasis. The successful application of patch-clamp technique on isolated vacuoles allows the determination of the functional characteristics of tonoplast ion channels and transporters. The parallel use of a sensor-based fluorescence approach capable of detecting changes in calcium and proton concentrations opens up new possibilities for investigation. In excised patch, the presence of fura-2 in the vacuolar solution reveals the direct permeation of calcium in plant TPC channels. In whole-vacuole, the activity of non-electrogenic NHX potassium proton antiporters can be measured by using the proton sensitive dye BCECF loaded in the vacuolar lumen by the patch pipette. Both vacuolar NHXs and CLCa (chloride/nitrate antiporter) are inhibited by the phosphoinositide PI(3,5)P_2_, suggesting a coordinated role of these proteins in salt accumulation. Increased knowledge in the molecular mechanisms of vacuolar ion channels and transporters has the potential to improve our understanding on how plants cope with a rapidly changing environment.

## Introduction

**Fig. 1:**
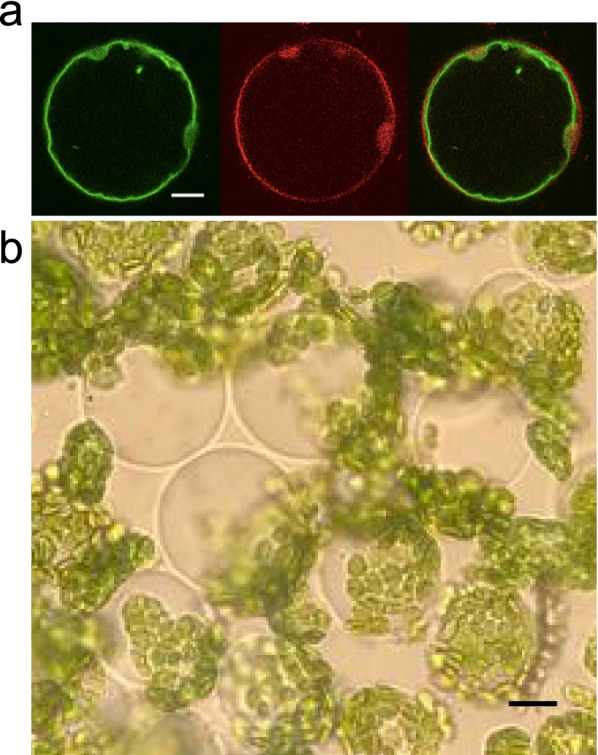
The plant vacuole can occupy most of the intracellular volume and is easy to isolate. **a** Confocal images showing an *Arabidopsis thaliana* mesophyll protoplast transiently expressing a tonoplast-localized AtTPC1-EGFP fusion protein (left, green signal) and stained with the plasma membrane marker FM4–64 (middle, red signal). The right panel displays merged signals. Scale bar 7 μm (see Supplemental material of Picco et al. 2015 for experimental details). **b** Protoplasts from Arabidopsis mesophyll cells were obtained by enzymatic treatment with cellulase and pectolyase (Scholz-Starke et al. 2006). Upon application of the vacuole release solution VRS (see text) they burst and release the vacuoles. Scale bar 10 μm

The vacuole is a peculiar compartment of plant cells, which in mature cells can occupy up to 90% of the cell volume, Fig. 1a. Despite it was originally considered to be the cell’s trash, we now know that it is a very versatile organelle (Eisenach et al. 2015). For example, a fundamental function of the vacuole is the storage of ions and molecules that can be mobilized in case of metabolic needs, when the plant is subjected to biotic or abiotic stress. The vacuole can be easily isolated from the cell in essentially two ways: mechanical excision and enzymatic treatment.

When a razor blade cuts a homogeneous tissue of the plant in thin slices, vacuoles are directly extruded to the recording chamber. This procedure was successfully applied to various plants and tissues, from sugar beet (Carpaneto et al. 1999b) and radish (Gambale et al. 1993) taproots to roots from the sweet-water pond plant *Eichhornia crassipes* (Paganetto et al. 2001) and leaves from the Mediterranean seagrass *Posidonia oceanica* (Carpaneto et al. 1997). In alternative, the cell wall surrounding the plant cell can be digested by treatment with specific enzymes such as cellulase, pectolyase and macerozyme. The experimental protocol, which was applied practically to all plant tissues, see Bregante et al. (1997) as an example of protoplast isolation from the root cortical tissue of *Zea mays*, and even to suspension-cultured cells (Costa et al. 2004), needs to be adapted to the selected plant preparation. The time needed for enzymatic treatment ranges from less than 1 h, as in the case of *Arabidopsis thaliana* mesophyll cells, up to more than 4 h for *Posidonia oceanica* leaves (Carpaneto et al. 2004). Exposing protoplasts to an ionic solution containing the calcium buffer ethylene glycol-bis(2- aminoethylether)-N,N,N′,N′-tetraacetic acid (EGTA) and having a reduced osmotic pressure usually induces a rupture of the plasma membrane and a consequent release of the internal vacuole. We found that the following Vacuole Release Solution containing (in mM): 100 malic acid, 155 Bis-tris-Propane (BTP), 5 EGTA, 3 MgCl_2_, 200 D-sorbitol, pH 7.33, is particularly effective in releasing vacuoles from protoplasts of Arabidopsis mesophyll cells, Fig. 1b (Costa et al. 2012); by filling a perfusion pipette with VRS and placing it in front of a intact protoplast, it is possible to release a single vacuole at a time (Hedrich 1995).

## The patch-clamp technique applied on isolated vacuoles

**Fig. 2:**
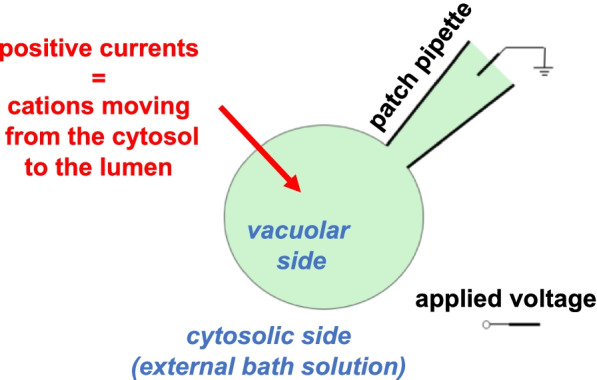
Cartoon of the patch-clamp technique applied on plant vacuoles. The patch clamp technique is applied in the whole-vacuole (cytosolic side-out) configuration. Positive currents correspond to the movement of cations from the cytosolic to the luminal side of the vacuole (or to the opposite movement of anions)

Vacuoles isolated both mechanically and by enzymatic treatment (see above) are an excellent preparation for the application of the patch-clamp technique. The most used configuration is whole-vacuole, Fig. 2. After placing a glass pipette with a diameter of a few micrometers on the tonoplast and applying a light suction, a strong contact is obtained between the tip of the pipette and the vacuolar membrane, with an electrical resistance which can exceed 5 GigaOhm. By simultaneously sucking and applying a fast (700–900 μs) high-voltage pulse (700–900 mV), the membrane subtended by the pipette can be broken: the solution inside the pipette perfuses inside the vacuolar lumen and, vice versa, the luminal solution is diluted inside the patch pipette. The washing of the vacuolar lumen depends on various parameters (Pusch and Neher 1988) such as access resistance (linked to the size and shape of the pipette), volume of the vacuole (which can be deduced from the measurement of the membrane capacity, proportional to the vacuolar surface), size of the molecules involved. Generally, the ions are able to diffuse inside/outside the vacuole in less than a minute.

It is interesting to observe that the cytosolic side of the vacuole faces the outside (or the bath solution), a reversed situation compared to what happens for a protoplast or an animal cell. To avoid confusion, the following convention has been adopted for endomembrane recordings (Bertl et al. 1992): the voltage is V_cyt_ -V_lumen_; positive currents correspond to the movement of positive charges from the cytosol to the vacuolar lumen (or to anions moving in the opposite direction). Therefore, from the electrical point of view, the outside of a cell is equivalent to the inside of the vacuole (and is grounded). Since the vacuoles do not adhere firmly to the bottom of the recording chamber, a perfusion system is required that combines efficiency in the change of solutions with great mechanical stability (Festa et al. 2016). Generally, by optimizing the recording chamber, by means of a gravity-driven system and using a peristaltic pump that withdraws the excess solution, it is possible to change the bath solution in about a minute and therefore study in detail the cytosolic factors capable of modulating the numerous channels or plant transporters present on the vacuolar membrane (Martinoia 2018).

It is also worth noting that the plant vacuole can be used to study the properties of toxins produced by plant pathogens (Carpaneto et al. 2002) or as a heterologous system for the study of lysosomal animal channels and transporters including human TPC channels (Festa et al. 2022), which share the same inhibitors (Benkerrou et al. 2019; Filippini et al. 2020) but not the same agonists (Boccaccio et al. 2014; Kirsch et al. 2018) with the plant homolog.

Concerning channels and transporters of the plasma membrane, in addition to the use of cell cultures, whose endogenous channels need to be studied (Carpaneto et al. 1999a), the heterologous system of reference for carrying out structure-function correlation studies is represented by Xenopus oocytes (Porée et al. 2005; Carpaneto et al. 2010; Derrer et al. 2013). Plant channels of the plasma membrane have also been successfully expressed in tobacco cells (Bregante et al. 2008).

## Potassium and calcium permeation in plant TPC channels

**Fig. 3:**
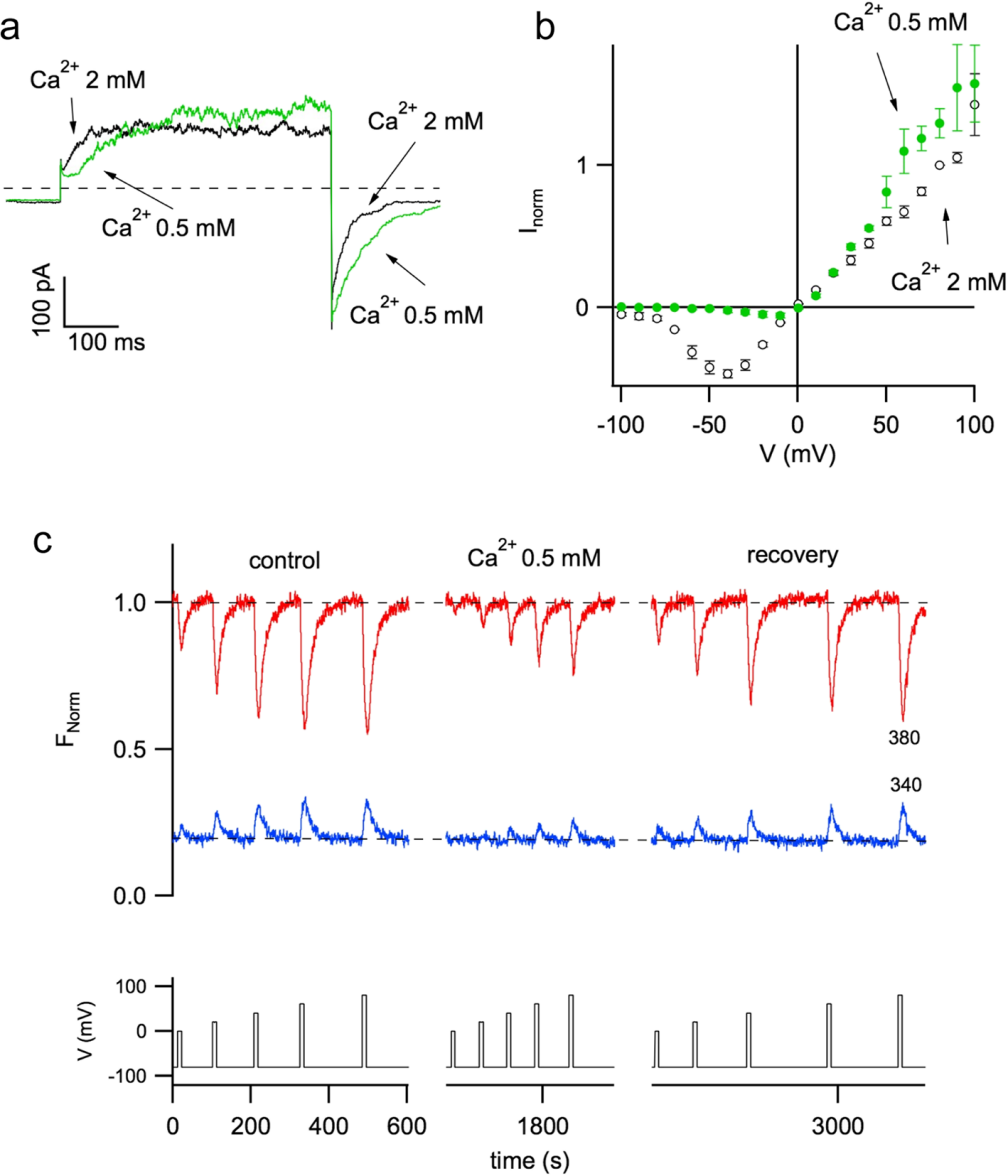
Plant TPC channels are modulated by cytosolic calcium. **a** Current recordings of carrot TPC channels in the presence of 2 mM (black trace) and 0.5 mM (green trace) cytosolic calcium. Main voltage pulse of + 80 mV; holding voltage was − 80 mV. **b** Stationary currents in 2 mM (black open circles) and 0.5 mM (green solid circles) cytosolic calcium were normalized to the value at + 80 mV in 2 mM Ca^2+^ and displayed versus voltage. Voltage pulses were ranging from − 100 mV to + 100 mV in 10 mV increments. **c** Patch pipette was filled with 100 μM of the calcium-sensitive dye fura-2. Fluorescence signals (upper panel) were induced by excitation light at 380 nm (red trace) and 340 nm (blue trace), respectively. The lower panel displayed 10 s voltage pulses of 0, + 20, + 40, + 60, + 80 mV, which were applied starting in 2 mM Ca^2+^ (control, left panel), at low calcium (Ca^2+^ 0.5 mM, middle panel), and again in 2 mM Ca^2+^ (recovery, left panel). Modification of Figs. 1 and 2, from Carpaneto and Gradogna (2018), Biophysical Chemistry, 236:1–7, reprinted by permission from Elsevier (license number 5333730921940)

From the whole vacuole configuration, by pulling the pipette it is possible to obtain the so-called cytosolic side-out excised patch configuration. In this configuration, only a small portion of the membrane is held by the pipette. If the density/conductance of the ion channels under investigation is sufficiently high, macroscopic currents can also be recorded in this mechanically more stable recording mode (Carpaneto and Gradogna 2018). In Fig. 3a the black trace corresponds to a current recorded in the presence of 2 mM cytosolic calcium, nanomolar concentrations of vacuolar Ca^2+^ and symmetrical concentrations of potassium (105 mM). A voltage pulse of + 80 mV, from a holding voltage of − 90 mV, activates the current, which reaches a steady state in about 100 ms. By applying a tail voltage of − 50 mV the current decays exponentially. If cytosolic calcium is decreased to 0.5 mM, current is only slightly affected, with a slowing down of both activation and deactivation time. However, the I-V relationship shifts of more than + 80 mV, Fig. 3b. The ion channels that mediate this type of currents have been named SV, namely Slow Vacuolar channels for their slow time of activation (Hedrich et al. 2018). It has been found that they are encoded by the *tpc* gene (Peiter et al. 2005). In addition to cytosolic calcium, these channels are modulated by many parameters such as magnesium (Pei et al. 1999; Carpaneto et al. 2001), redox agents (Scholz-Starke et al. 2005) and polyunsaturated fatty acids (Gutla et al. 2012).

Since the physiological voltage of the vacuole ranges from − 30  to 0 mV (Hedrich 2012), from Fig. 3b it can be deduced that plant TPC channels are closed under physiological conditions. The factor that can move the channel activation curve towards more negative physiological voltages (Pottosin et al. 1997) is not yet known, despite new insights gained from the recent cryoEM structures of the plant TPC1 channel (Ye et al. 2021; Dickinson et al. 2022).

Currents of Fig. 3 are essentially due to the movement of potassium (Hedrich and Neher 1987). If the calcium sensitive dye fura-2 is added to the pipette solution, application of positive voltages induces fluorescence changes that are compatible with the movement of calcium from the cytosol to the vacuolar lumen, Fig. 3c left panel. These signals are completely absent if the experiments are carried out on vacuoles from Arabidopsis mutants lacking the endogenous TPC (Gradogna et al. 2009). These recordings therefore represents a direct demonstration of the calcium permeability of the plant TPC, validated by recent structure-function studies (Guo et al. 2017); however, the issue of calcium permeation in plant TPC channels under physiological conditions is still controversial (Navarro-Retamal et al. 2021). When cytosolic calcium is lowered, fluorescence signals are significantly reduced, Fig. 3c left panel. It should be noted that at high positive voltages no significant variation of the currents was noticeable upon cytosolic calcium reduction (Fig. 3a). The effects are completely reversible, right panel of Fig. 3c (recovery). By performing an appropriate calibration (Gradogna et al. 2009; Carpaneto and Gradogna 2018), it was possible to estimate the relative contribution to the TPC current of calcium and potassium; from voltages between + 60 and + 80 mV the ratio between the total current and the calcium current increased from about 10 at Ca^2+^ = 2 mM to about 20 at Ca^2+^ = 0.5 mM (Carpaneto and Gradogna 2018). The use of a different approach, namely the MIFE technique, yielded similar results as demonstrated by Pérez et al. (2008), see Pottosin and Dobrovinskaya (2022) for a comprehensive review on plant TPC channels.

**Fig. 4:**
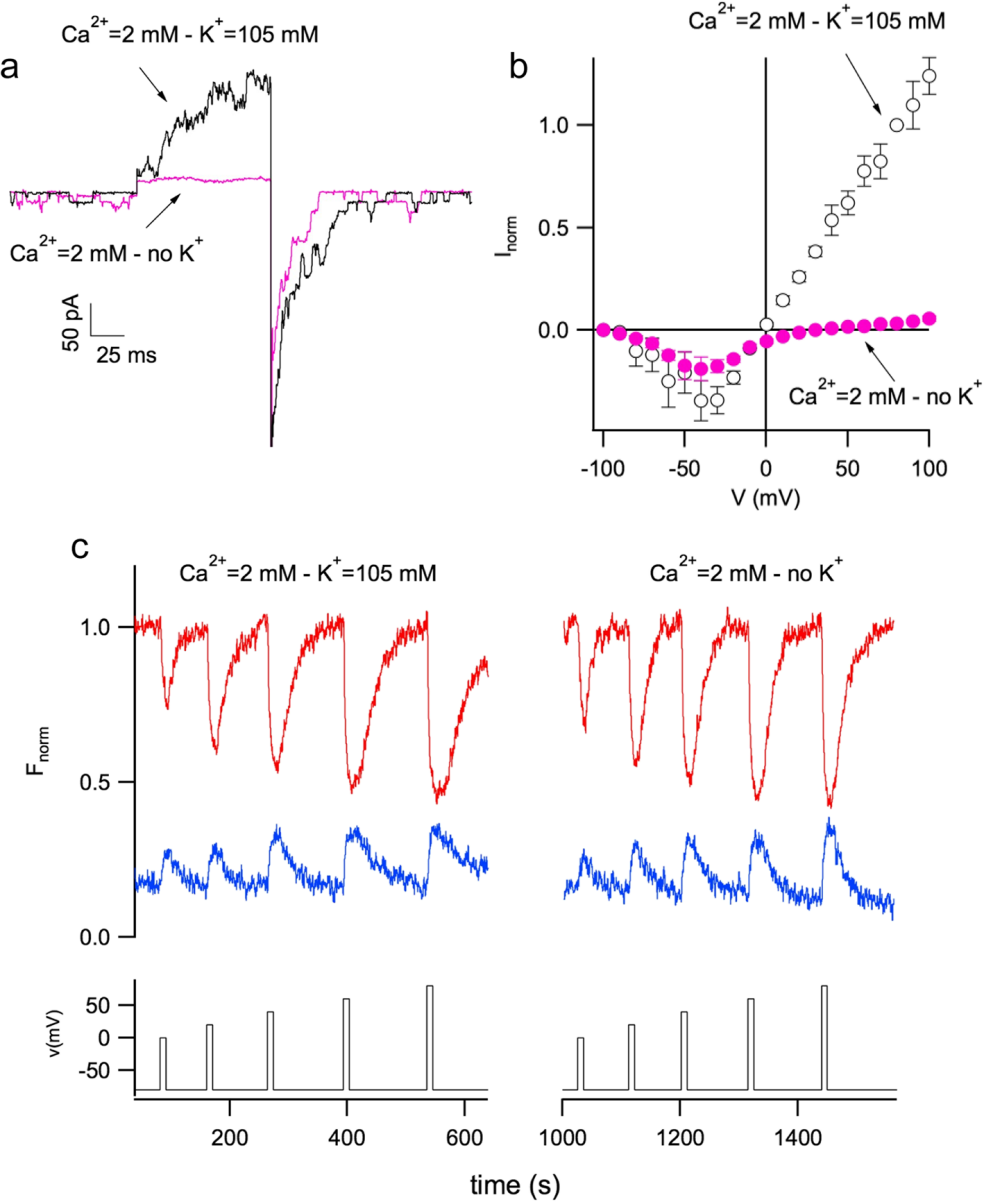
Removal of cytosolic potassium strongly reduced TPC currents but did not change calcium permeation. **a** Currents of carrot TPC channels recorded in control condition (Ca^2+^ = 2 mM – K^+^ = 105 mM, black trace) and in the absence of cytosolic K^+^ (Ca^2+^ = 2 mM – no K^+^, violet trace). Main voltage pulse of + 80 mV lasting 100 ms. Holding and tail voltage of − 80 mV. **b** Stationary currents (normalized to the value at + 80 mV in control) in the presence (open black circles) and absence of cytosolic potassium (solid violet circles) were plotted versus applied voltage. **c** In the upper panel, fura-2 signals did not change significantly in control condition (Ca^2+^ = 2 mM – K^+^ = 105 mM, left panel) and upon removal of cytosolic potassium (Ca^2+^ = 2 mM – no K^+^, right panel). The lower panel displays the applied voltage (10 s voltage pulses from 0 mV to + 80 mV, in 20 mV steps, from a holding potential of − 80 mV). Modification of Figs. 4 and 5, from Carpaneto and Gradogna (2018), Biophysical Chemistry, 236:1–7, reprinted by permission from Elsevier (license number 5333730921940)

When cytosolic potassium is removed, the effect on the currents elicited by positive voltages is dramatic as shown in Fig. 4a. At negative voltage, on the other hand, changes are not significant, Fig. 4b, an indication that cytosolic potassium does not modify the voltage dependence of the channel. Interestingly, at positive voltages, the removal of potassium has a negligible effect on the permeation of calcium as it is evident from the fluorescence signals presented in the left panel of Fig. 4c.

Overall, these experiments indicate that the fluorescence approach combined with the classic electrophysiological recordings allows a more accurate functional characterization of the channel.

## A novel approach for investigating the activity of NHX transporters

**Fig. 5:**
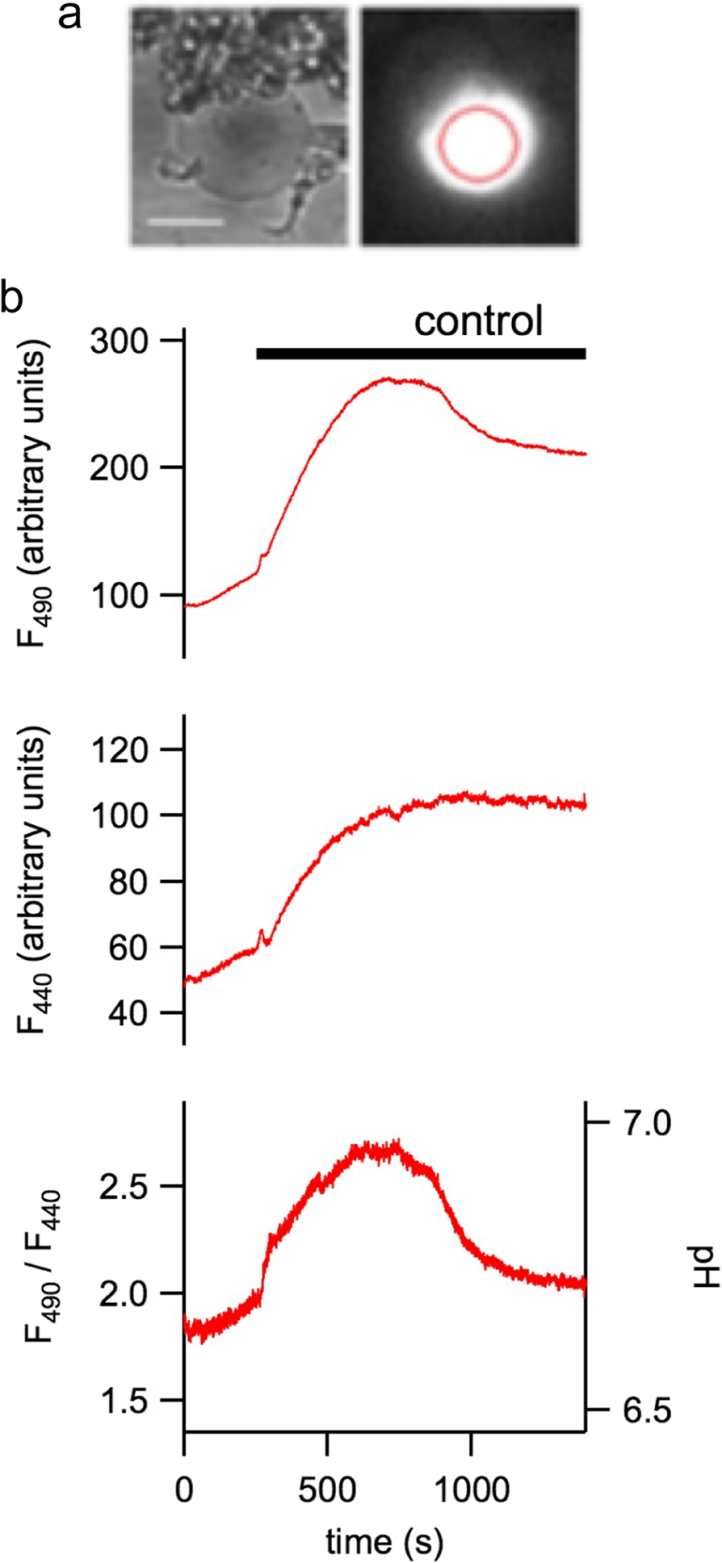
The loading phase of the vacuole with the proton sensitive fluorophore BCECF. **a** The left panel shows a bright-field image of a micropipette placed on an isolated vacuole. Scale bar, 10 μm. The whole-vacuole configuration allows the loading of the fluorophore inside the vacuolar lumen. In the right panel the fluorescence image of the same vacuole was obtained with a 490 nm excitation light and detected using a 515-nm bandpass emission filter. The red circle is the region of interest (ROI) where fluorescence is evaluated. **b** After establishment of the whole-vacuole configuration it is possible to follow the time course of BCECF fluorescence emission signals, F490 (excitation at 490 nm, upper panel), F440 (excitation at 440 nm, middle panel) and fluorescence ratio (F490/F440 and pH, lower panel). Seal and break-in are obtained in VRS, i.e. the solution able to blast the protoplast, which is changed to control bath solution after about 250 s. Modification of Fig. S2 and S4, from Gradogna et al. (2021), New Phytologist, 229:3026–3036, reprinted by permission from John Wiley and Sons (license number 5333730373195)

The left panel of Fig. 5a shows a vacuole, isolated from the mesophyll of Arabidopsis, together with a patch pipette, visible on the right, resting on the tonoplast membrane. The pipette ionic solution contains the proton sensitive dye 2′,7′-Bis(2-carboxyethyl)-5(6)-carboxyfluorescein (BCECF) at a concentration of 10 μM. The whole-vacuole configuration allows the fluorophore to enter the vacuolar lumen as observed in the image of Fig. 5a right panel, obtained by collecting emission light at 515 nm after having excited the same vacuole in the left panel with light at 490 nm. The loading characteristic of the fluorophore is shown in Fig. 5b. The signals at excitation light respectively of 440 and 490 nm increase after t=0 s, the time in which the break-in occurred, i.e. the piece of membrane subtended by the pipette after the seal is broken in order to reach the whole-vacuole configuration. In this experiment, about 20 minutes have elapsed before the ratio between the two wavelengths is stable, bottom panel of Fig. 5b. In general, the loading phase can last from 10 to 30 minutes depending on the size of the vacuole and the access resistance of the pipette. The access resistance in turn depends on the size and shape of the tip, the concentration of salts in the pipette solution and the quality/stability of the break in.

**Fig. 6:**
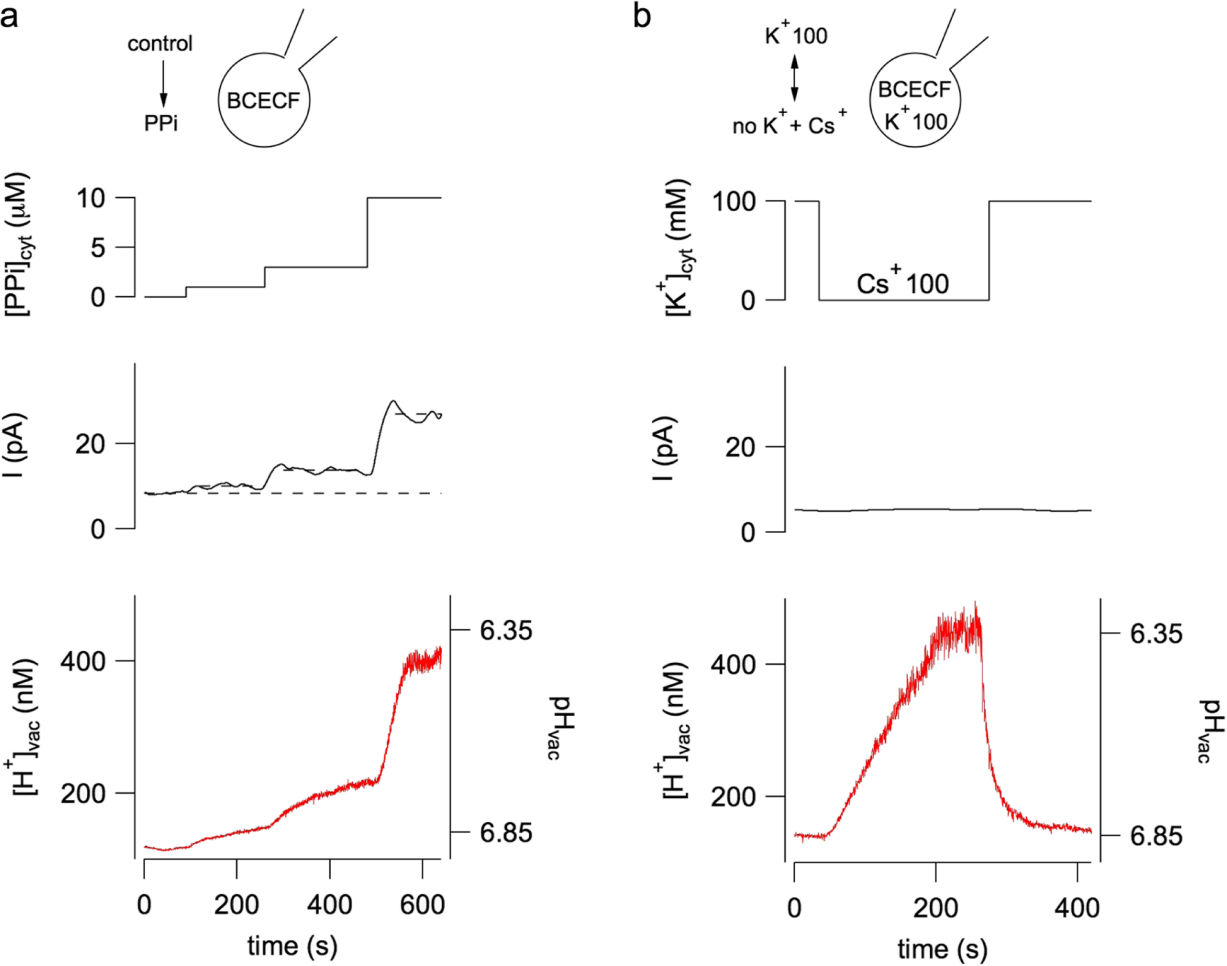
Vacuolar acidification revealed by the proton-sensitive fluorescent dye BCECF. **a** Inorganic pyrophosphate (PPi) is added to the bath solution (top panel) at concentrations of 1, 3 and 10 μM (dotted lines indicate that the switching of the bath solution is irrespective to the real change due to the perfusion system, see Gradogna et al. 2021 for a discussion about the effects of the perfusion). Middle panel shows the correspondent time course of tonoplast membrane current due to vacuolar proton-pumping pyrophosphatase activation. Bis(2-carboxyethyl)-5(6)-carboxyfluorescein (BCECF) loaded inside the vacuole through the patch pipette allows the determination of luminal proton concentration changes (bottom panel). **b** Substitution of cytosolic bath solution potassium with an equimolar amount of caesium ions is schematically displayed in the top panel. The middle panel shows that there is no change in background current (holding voltage of 0 mV). However a significant acidification of the luminal solution is apparent (bottom panel). Modification of Fig. 1, from Gradogna et al. (2021), New Phytologist, 229:3026–3036, reprinted by permission from John Wiley and Sons (license number 5333730373195)

If pyrophosphate is added to the cytosolic bath solution once the loading phase is complete, currents mediated by vacuolar pyrophosphatase (V-PPiase) can be measured, Fig. 6a central panel. The presence of the BCECF inside the vacuole allows to detect the corresponding passage of protons from the cytosol to the vacuolar lumen as shown in the lower panel of Fig. 6a. Changes in both current and vacuolar pH are dependent on the concentration of pyrophosphate. These experiments allow us to evaluate the ability of the fluorophore to detect changes in protons: in our experimental system even very small currents result in particularly significant pH changes.

**Fig. 7:**
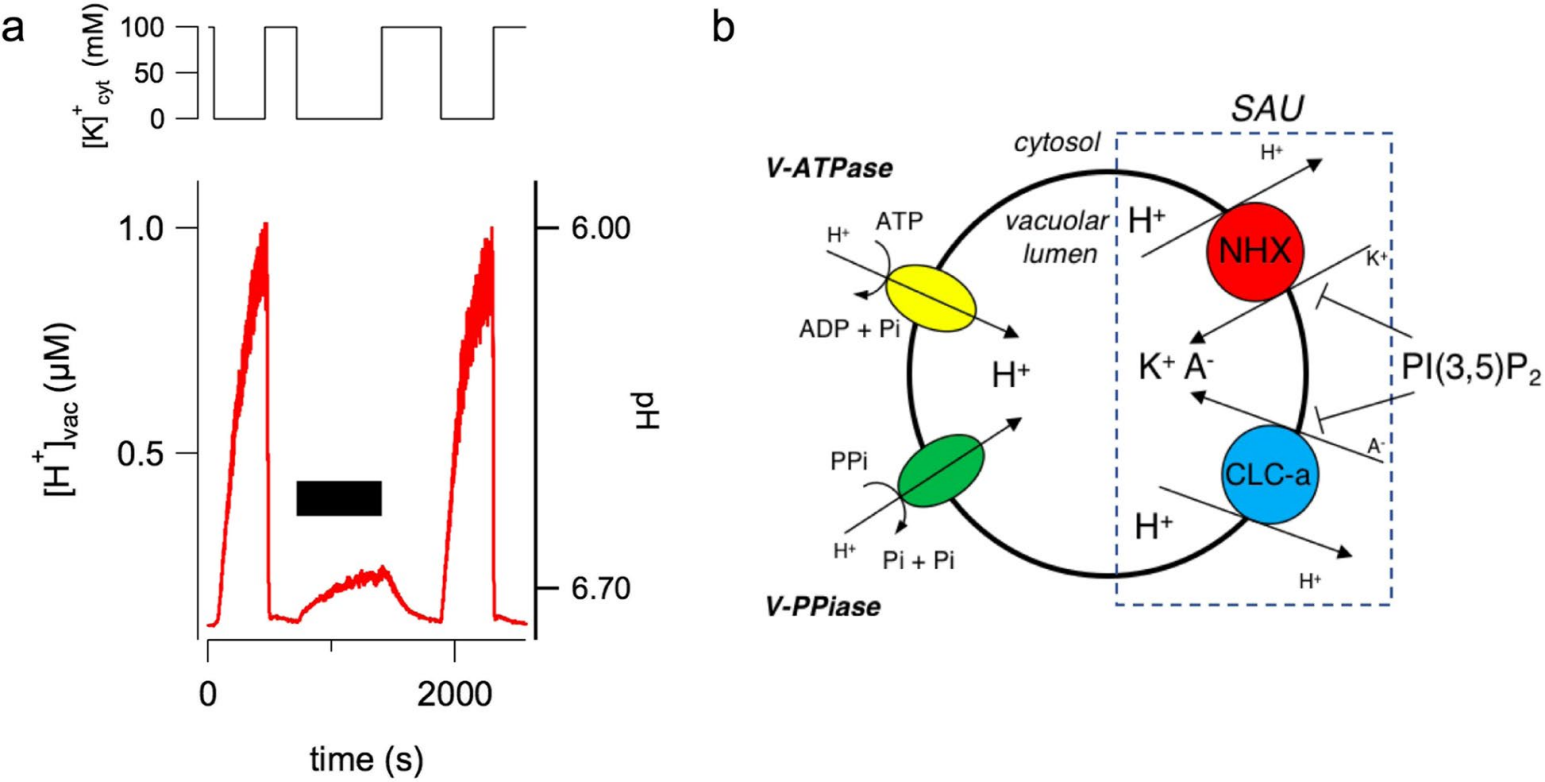
The phosphoinositide PI(3,5)P_2_ inhibits NHX activity. **a** PI(3,5)P_2_ added at a concentration of 200 nM in the bath solution lacking potassium, which is substituted by the large, membrane- impermeable cation BTP^+^ (no K^+^ + BTP^+^), induces a strong and reversible inhibition of the vacuolar acidification mediated by NHX activity. **b** Scheme of the tonoplast key players responsible of vacuolar salt uptake. A Salt Accumulation Unit (SAU) is formed by NHXs together with CLC-a. V-ATPase, vacuolar H^+^-ATPase; V-PPiase, vacuolar H^+^- pyrophosphatase. A^−^, H^+^ and K^+^ indicates respectively anions, protons and potassium ions; the dimension of the letters for the ions is proportional to their concentration. Modification of Fig. 6, from Gradogna et al. (2021), New Phytologist, 229:3026–3036, reprinted by permission from John Wiley and Sons (license number 5333730373195)

Pyrophosphate is not the only way to vary the pH inside the vacuole. If potassium is replaced in the cytosolic solution with an equivalent concentration of the cesium ion, Fig. 6b top panel, no current variation is observed, Fig. 6b central panel. However, BCECF detects a very significant increase in proton concentration. This increase is completely absent in vacuoles isolated from Arabidopsis knockout plants for NHX1/2 transporters (Gradogna et al. 2021). Therefore, this approach is able to detect the activity of non-electrogenic transporters and to investigate their possible modulators. In Fig. 7a the addition of 200 nM phosphatidylinositol-3,5-bisphosphate (PI(3,5)P_2_) to the cytosolic bath solution reversibly inhibits NHX transporters. PI(3,5)P_2_ is a low-abundance signaling lipid associated with the tonoplast in plant cell and with endo-lysosomal membranes in eukaryotic cells (Balla 2013). It is very interesting to note that PI(3,5)P_2_ is able to inhibit also another vacuolar transporter, CLCa (Carpaneto et al. 2017), as shown schematically in Fig. 7b. The vacuolar membrane has two proton pumps, the V-ATPase and the V-pyrophosphatase, which generate a proton motive force with two components. The main component is the proton gradient between the cytosol and the vacuolar lumen with a concentration of protons inside the vacuole that is at least two orders of magnitude higher. The secondary component is the tonoplast voltage of about − 30 mV, as mentioned, which also favors the movement of protons from the inside of the vacuole to the outside. The proton motive force generated by the pumps is used by the two antiporters, NHX and CLCa, to move respectively potassium and anions (nitrate and chlorine) towards the vacuolar interior. The combined action of the two antiporters therefore tends to increase the concentration of salts within the vacuolar lumen. The fact that both proteins are inhibited by PI(3,5)P_2_ defines a Salt Accumulation Unit (SAU), whose activity needs to be minimized in case of release of salts. In support of this hypothesis, in the closing mechanism of the stomata the concentration of PI(3,5)P_2_ increases (Bak et al. 2013) and, through the inhibition of NHX and CLCa, favors the release of salts and the decrease of cellular turgor.

## Conclusions

The application of the patch-clamp technique on isolated vacuoles is very useful for the functional characterization of vacuolar channels and transporters, which play a fundamental role in plant physiology. The extension of the technique with fluorescence methods opens up new possibilities such as the study of the activity of non-electrogenic transporters. Other approaches, based on advanced electrophysiological techniques combined with genetically encoded sensors, are recently emerging (Dindas et al. 2021). This will increase our knowledge on how plants work and suggest new strategies to improve crop productivity in case of biotic and abiotic stress.

## Data Availability

Not applicable.

## Abbreviations

BCECF: 2′,7′-Bis(2-carboxyethyl)-5(6)-carboxyfluorescein
EGTA: 5 ethylene glycol-bis(2- aminoethylether)-N,N,N′,N′-tetraacetic acid
BTP: Bis-tris-Propane
PI(3,5)P_2_: Phosphatidylinositol-(3,5)-bisphosphate
V-PPiase: Vacuolar pyrophosphatase
